# Dynamic distress calls: volatile info chemicals induce and regulate defense responses during herbivory

**DOI:** 10.3389/fpls.2023.1135000

**Published:** 2023-06-19

**Authors:** Nithya N. Kutty, Manasi Mishra

**Affiliations:** Department of Biosciences and Technology, Dr. Vishwanath Karad MIT-World Peace University, Pune, Maharashtra, India

**Keywords:** herbivory, defense responses, VOCs (volatile organic compounds), HIPVs, terpenoids, biotic stress, metabolomics

## Abstract

Plants are continuously threatened by a plethora of biotic stresses caused by microbes, pathogens, and pests, which often act as the major constraint in crop productivity. To overcome such attacks, plants have evolved with an array of constitutive and induced defense mechanisms— morphological, biochemical, and molecular. Volatile organic compounds (VOCs) are a class of specialized metabolites that are naturally emitted by plants and play an important role in plant communication and signaling. During herbivory and mechanical damage, plants also emit an exclusive blend of volatiles often referred to as herbivore-induced plant volatiles (HIPVs). The composition of this unique aroma bouquet is dependent upon the plant species, developmental stage, environment, and herbivore species. HIPVs emitted from infested and non-infested plant parts can prime plant defense responses by various mechanisms such as redox, systemic and jasmonate signaling, activation of mitogen-activated protein (MAP) kinases, and transcription factors; mediate histone modifications; and can also modulate the interactions with natural enemies *via* direct and indirect mechanisms. These specific volatile cues mediate allelopathic interactions leading to altered transcription of defense-related genes, *viz.*, proteinase inhibitors, amylase inhibitors in neighboring plants, and enhanced levels of defense-related secondary metabolites like terpenoids and phenolic compounds. These factors act as deterrents to feeding insects, attract parasitoids, and provoke behavioral changes in plants and their neighboring species. This review presents an overview of the plasticity identified in HIPVs and their role as regulators of plant defense in Solanaceous plants. The selective emission of green leaf volatiles (GLVs) including hexanal and its derivatives, terpenes, methyl salicylate, and methyl jasmonate (MeJa) inducing direct and indirect defense responses during an attack from phloem-sucking and leaf-chewing pests is discussed. Furthermore, we also focus on the recent developments in the field of metabolic engineering focused on modulation of the volatile bouquet to improve plant defenses.

## 1 Introduction

Plants are sessile organisms that are very often exposed to several biotic and abiotic stresses during their lifetime. To cope with adverse conditions, plants produce an array of organic compounds called secondary or specialized metabolites (Tisser et al., 2014). Specialized metabolites can be defined as those metabolites that are directly or indirectly involved in plant reproduction, development, defense, and help in mediating ecological interactions with the environment (Tisser et al., 2014). These are diverse compounds that may or may not be produced in all parts of the plant. Accumulation of specialized metabolites also vary from plant species to species, thus contributing to the diversity of these compounds (Knudsen et al., 2006; Dudareva et al., 2013). Based on the chemical structures, they can be broadly classified as polyphenols (lignin, flavonoids, and phenolic acids), nitrogen-/sulfur-containing compounds (alkaloids, glucosinolates, and thiophenes), and terpenoids (including carotenoids) (Tisser et al., 2014). Specialized metabolites also include volatile organic compounds (VOCs), which are low molecular weight molecules with low water solubility and high vapor pressure (Muhlemann et al., 2014). VOCs mediate several functions and act as chemical messengers during different biotic/abiotic stress situations through their elevated levels of secretion and emission (Muhlemann et al., 2014). Biotic stress affecting plants includes attacks from herbivores and infections from bacteria, fungi, and viruses (Suzuki et al., 2014). Herbivores can be further classified based on their attacking mode into chewing herbivores, which cause more cellular damage; mesophyll-feeding stylet feeders; and phloem-feeding stylet feeders, which cause less structural damage to plant tissue but can cause the source to sink shifts by emptying the cellular contents (Kant et al., 2009). This review focuses on how volatile compounds are produced dynamically within the family Solanaceae under the attack of herbivores. The diversity of VOCs produced by Solanaceous crops constitutively and upon attack by different classes of herbivores is also discussed in detail. Secretion and emission of VOCs from these crops during pest attacks can also be affected by abiotic factors (Vázquez-González et al., 2022). However, the influence of these factors affecting plant defense is beyond the scope of this review and have limited to biotic stresses. Furthermore, an attempt is made towards understanding the mechanism of volatile-induced selective defense strategies in these crops. Solanaceous crop plants are considered as most important crops for not only fulfilling the nutritional requirements of vegetables but also as a source of drugs, ornamentals, and medicines (Yadav et al., 2016). Solanaceous plants are attacked by major plant pathogens and pests like bacteria, fungi, nematodes, oomycetes, parasites, and lepidopteran insect pests (Strange and Scott, 2005). Solanaceous plants are known for their distinct evolutionary history driven by natural selection resulting into unique adaptations, signaling molecules, and biochemical pathways involving several genes and products (Poczai et al., 2022). Consequently, they have served as important model plants for studying plant defense mechanisms. We further highlight the ecological importance of these defense mechanisms in the Solanaceae family concerning different types of pests and conclude by listing the possible modifications for improved pest resistance through metabolic engineering in Solanaceous crops.

## 2 Herbivore- induced plant VOCs

Approximately 1,700 VOCs have been identified from different plant species (Dudareva et al., 2006). Based on their core chemical structure and biosynthesis pathways, VOCs are classified as terpenoids, benzenoids/phenylpropanoids, fatty acid derivatives, and amino acid derivatives (Dudareva et al., 2006). These compounds were initially considered metabolic waste products but were eventually discovered to have important physiological roles (Harrewijn and Piron, 1995). Plants produce VOCs from almost all parts, including leaves, roots, flowers, and fruits into the atmosphere (Dudareva et al., 2006). Among these plant parts, floral volatiles constitute almost 90% of the total mixture of compounds. Floral VOCs play a major role in the attraction of pollinators and thereby help in plant reproduction (Dudareva et al., 2006). Plants also emit VOCs from vegetative tissues, which are normally elevated under biotic/abiotic stress conditions (Dicke and Baldwin, 2010). These compounds also mediate plant defense and carry out tri-trophic and allelopathic interactions. Root volatiles play a major role in defense against nematodes and are actively involved in plant–microbe interactions at the underground level (Huang et al., 2019). The emitted aerial VOCs constitute the headspace of the plants. Along with the emitted bouquet of VOCs, these compounds can be secreted and stored as glycosyl-bound volatiles in plant tissues (Song et al., 2018). The emission of VOCs is influenced by different physical factors, including the circadian clock, light, temperature, and humidity (Dudareva et al., 2006). The emission rate of VOCs is also influenced by the developmental stage of the plant, environmental factors, and presence/absence of abiotic/biotic stress elements (Dudareva et al., 2006).

**Figure 1:**
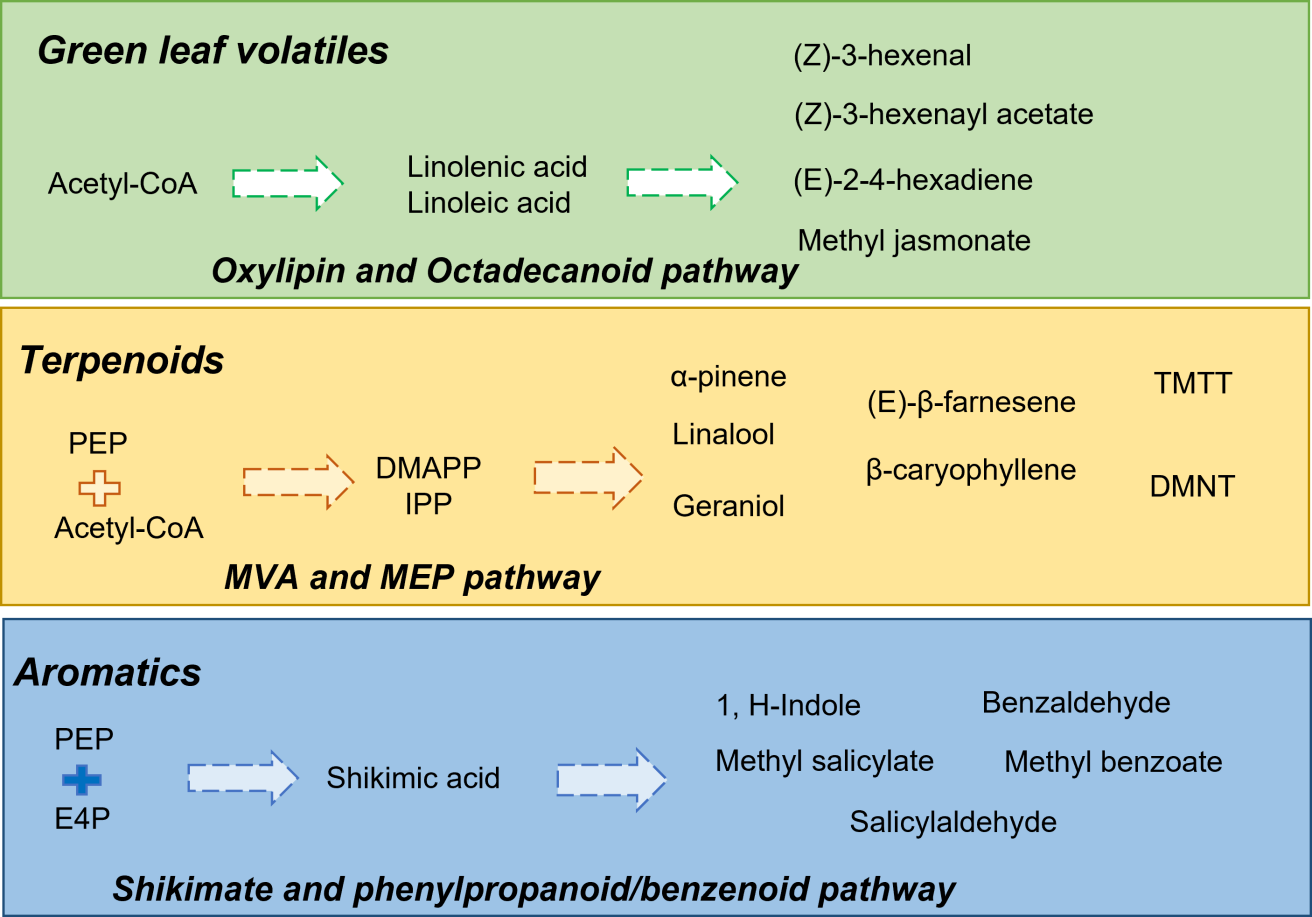
Overview of different classes of herbivore induced plant volatiles (HIPVs), including green leaf volatiles, terpenoids, and aromatic compounds along with their biosynthesis pathways. In the figure, block arrows represent multiple biosynthetic steps involved. Abbreviations: acetyl-CoA, acetyl-coenyzme A; DMAPP, dimethylallyl pyrophosphate; DMNT, 4,8-dimethylnona-1,3,7-triene; E4P, erythrose 4-phosphate; IPP, isopentenyl pyrophosphate; PEP, phosphoenolpyruvate; TMTT, 4,8,12-trimethyltrideca-1,3,7,11-tetraene.

Herbivore-induced plant volatiles (HIPVs) refer to the class of VOCs that are specifically released by plants during herbivory (Dicke et al., 2009). Several HIPVs have been identified from almost 200 families of plants (War et al., 2011). These compounds may be released by the infected tissues or uninfected tissues, thereby leading to an altered scent profile of the plant. Furthermore, HIPVs act as information molecules conveying pest attacks in host plants to parasitoids/predators and other neighboring plants (Unsicker et al., 2009). Along with acting as info chemicals, these VOCs also increase the defense responses in plants (Mumm and Dicke, 2010). In recent times, several articles have been published regarding the diversity of HIPVs. These include the major classes, including GLVs, terpenoids, phenylpropanoids/benzenoids, and even small molecules like methanol and ethylene (Mumm and Dicke, 2010). The biosynthesis of green leaf volatiles (GLVs) or fatty acid derivative compounds is initiated by the deacetylation of galactolipids to free linolenic acid and linoleic acid in the plastids (Feussner and Wasternack, 2002). This is followed by the enzymatic activities of lipoxygenases, alcohol dehydrogenases, and alcohol acyl transferases, leading to the production of C_6_ alcohols, aldehydes, and esters (Kim and Grosch, 1981; Matsui et al., 1996; Grechkin, 1998; Howe et al., 2000). Linolenic acid also acts as a precursor compound for the synthesis of jasmonic acid (JA), which is further converted to methyl jasmonate (MeJA) and *cis*-jasmone (Seo et al., 2001; Song et al., 2005). Another important class of HIPVs includes terpenoid classes of isoprene (C_5_), monoterpenes (C_10_), sesquiterpenes (C_15_), and irregular terpenes. The diversity of terpenoid compounds is due to terpene synthases, which produce these compounds from two inter convertible C5 units, isopentenyl diphosphate (IPP) and its allelic isomer dimethylallyl diphosphate (DMAPP) (McGarvey and Croteau, 1995; Bohlmann et al., 1998; Dudareva et al., 2003; Sharkey et al., 2005). The biosynthesis pathways of terpenoids are also compartmentalized in plastids and cytosol (Poulter et al., 1981). The two terpenoid pathways, the mevalonic acid (MVA) pathway (sesquiterpenes synthesis) and the methylerythritol phosphate (MEP) pathway (monoterpenes synthesis), and their regulatory factors have been studied in model systems like *Petunia hybrida*, *Arabidopsis thaliana*, and many other species (Dudareva et al., 2004). Phenylpropanoid/benzenoid volatiles are derived from the aromatic amino acid, phenylalanine (Humphreys and Chapple, 2002). Like terpenoid compounds, extensive studies have been done to understand their biosynthesis pathways and regulatory factors. Benzenoid volatiles are synthesized from phenylalanine originating *via* the shikimate–chorismate pathway and followed by the deamination of phenylalanine to cinnamic acid by phenylalanine ammonia-lyase (PAL) (Vogt, 2010). A few of the volatiles under this class include methyl benzoate, methyl salicylate, benzyl alcohol, benzyl benzoate, benzyl salicylate, eugenol, and isoeugenol (Vogt, 2010; Muhlemann et al., 2014). Nitrogen-containing compounds such as 1H-indole and methyl-2-amino benzoic acid are also produced from the shikimate pathway (Muhlemann et al., 2014). A brief overview of select classes of HIPVs along with their biosynthetic routes is illustrated in **Figure 1**.

The diversity of these VOCs enables the plants to produce specific cues under biotic stress such as herbivory. The production of these volatile compounds during herbivory can be either triggered by tissue damage or damage-associated molecular patterns (DAMPs) or specific elicitors released by the pests referred to as herbivore-associated molecular patterns (HAMPs) (Meents and Mithöfer, 2020). The former is referred to as damage-induced volatiles (DIVs), while the latter is often referred to as herbivore-induced plant volatiles (HIPVs). Over the years, most of the studies have been taken up on HIPVs as compared to DIVs (Meents and Mithöfer, 2020). Recent studies have reported that mechanical damage inflicted on the plant can trigger systemic responses and herbivore resistance (Quintana-Rodriguez et al., 2018; Heil, 2009; Duran-Flores and Heil, 2016). Here, we try to identify the dynamic plasticity of the emitted volatiles from aerial tissues of Solanaceae crops under herbivory and the consecutive molecular responses related to defense and tri-trophic interactions.

## 3 Aroma compounds of Solanaceae plants during herbivory

The Solanaceae family includes flowering plants, many of them being economically important crops related to horticulture (petunia), pharmacology (tobacco, mandrake, and jimsonweed), and food (eggplant, potato, tomato, cherry, and gooseberry) (Samuels, 2015). According to the Food and Agricultural Organization (FAO) report, potatoes ranked sixth in the production of primary crops almost accounting for 359,071 thousand tons in 2022 (https://www.fao.org/3/CC2211EN/online/CC2211EN.html#). This also indicates the economic importance of these crops worldwide. One of the major problems faced by these crops is the attack from pests and pathogens. According to FAO, pests account for 20%–40% of the yield losses worldwide (https://www.fao.org/3/CC2211EN/online/CC2211EN.html#). These crops are affected by a variety of herbivores, including chewing herbivores, mesophyll feeding stylet feeders, and phloem-feeding stylet feeders (Kant et al., 2009). In the following sections, we describe the diversity of VOCs produced under the attack of major pests affecting agriculturally important Solanaceae crops (**Table 1**).

**Table 1:** Diversity of herbivore-induced plant volatiles in Solanaceae crops.

S. No.		Plant	Herbivore	Response of HIPVs					References	
				Terpenoids	GLVs		Phenylpropanoids/benzenoids			
Chewing herbivores										
1		*Nicotiana attenuata*	*Manduca sexta*	+	+		–		Halitschke et al., 2008	
2		*Solanum lycopersicum*	*Spodoptera exigua*	+	+		+		Zebelo et al., 2014; Disi et al., 2017	
3		*Solanum tuberosum*	*Leptinotarsa decemlineata* (Say)	N.D.	N.D.		N.D.		Landolt et al., 1999	
4		*Solanum tuberosum* *Solanum lycopersicum*	*Tuta absoluta*	+	–		–		Chen et al., 2021	
5		*Nicotiana tobaccum*	*Helicoverpa assulta* (Guene´e)	+	+		–		Sun et al., 2012	
6		*Nicotiana tobaccum*	*Phthorimaea operculella*	–	+		–		Xiang et al., 2020	
7		*Solanum lycopersicum*	*Tuta absoluta*	+	+		+		Silva et al., 2017	
8		*Capsicum annuum*	*N.M*		+				Freundlich et al., 2021	
9		*Solanum melongena*	*Leucinodes orbonalis* Guenee	+	+		+		Nusra et al., 2021	
10		*Solanum lycopersicum*	*Tuta absoluta*	+	+		+		Ayelo et al., 2021	
11		*Solanum tuberosum*	*Leptinotarsa decemlineata (Say)*	+	+		+		Davidson-Lowe, 2021	
12		*Solanum tuberosum*	*Leptinotarsa decemlineata (Say)*	+	+		–		Gosset et al., 2009	
13		*Solanum tuberosum*	*Mamestra brassicae L.*	+	+		+		Schettino et al., 2017	
Mesophyll-feeding stylet feeders										
14		*Capsicum annuum*	*Tetranychus urticae*	+	+		–		Zhang et al., 2020	
15		*Solanum lycopersicum*	*Tetranychus urticae*	+	–		+		Kant et al., 2004; Weinblum et al., 2021	
16		*Nicotiana tobaccum*	*Frankliniella occidentalis*	+	–		–		>Delphia et al., 2007	
17		*Solanum lycopersicum*	*Frankliniella occidentalis*	+	–		–		Escobar-Bravo et al., 2017; Chen et al., 2018	
18		*Lycopersicon esculentum*	*Tetranychus urticae*	+	–		–		van Schie et al., 2007; Falara et al., 2014	
19		*Solanum sarrachoides Sendtner, S. villosum Miller and S. scabrum Miller*	*Tetranychus evansi*	–	+		–		Murungi et al., 2016	
20		*Nicotiana tabacum, Solanum melalonga, Datura stramonium, Capsicum annuum*	*Tetranychus urticae* Koch	+	+		+		Van Den Boom et al., 2004	
21		*Solanum melongena L.*	*Frankliniella occidentalis (Pergande)*	N.D.	N.D.		N.D.		Liu et al., 2022	
22		*Capsicum annuum and Capsicum chinense*	*Frankliniella occidentalis*	+	–		–		Macel et al., 2019	
Phloem-feeding stylet feeders										
23		*Solatium berthaultii*	*Myzus persicae (Sulzer)*	+	–		–		Gibson and Pickett, 1983	
24		*Solanum tuberosum*	*Myzus persicae*	+	–		–		Harmel et al., 2007	
25		*Solanum melongena L.* *Capsicum annuum*	*Myzus persicae*	+	+		+		Digilio et al., 2012; Ali et al., 2022	
27		*Solanum pennellii LA716 × Solanum lycopersicum ‘Moneyberg’*	*Besmia tabaci*	+	–		–		Bleeker et al., 2009; Bleeker et al., 2011	
28		*Solanum lycopersicon L.* cv. *Moneymaker*	*Besmia tabaci*	+	–		+		Silva et al., 2017	
29		*Solanum lycopersicum*	*Trialeurodes vaporariorum*	+	–		–		Ayelo et al., 2021	

'+': Emission of compound; '-': absence of emission of compound. ND, Not determined

### 3.1 HIPVs and DIVs during attack of chewing herbivores

Chewing herbivores such as caterpillars, miners, and borers cause more harm to the plant tissues than the sucking pests. These pests directly damage the cell membranes and cell walls. Along with the tissue damage, it has been observed that chewing herbivores trigger defense responses, including the emission of volatiles *via* effectors. Effectors are molecules that can uplift/trigger defense responses in host plants. They include oral secretions or regurgitant, saliva, ventral eversible gland secretions, waste products, ovipositional fluids, and herbivore-associated endosymbionts (Kant et al., 2009). Chemically, they can be fatty acid conjugates (e.g., volicitin), β-glucosidases, and small peptide molecules (Mattiacci et al., 1995; Alborn et al., 1997; Mori et al., 2001). The detection of the presence of volicitin and other volatile-inducing fatty acid conjugates in oral secretions of tobacco budworm and tobacco hornworm is one of the earliest reports in Solanaceae crops (Halitschke et al., 2001; Mori et al., 2001). Ventral eversible gland secretions of *Spodoptera exigua* caterpillars have also been shown to significantly increase the production and emission of GLVs ((*E*)-2-hexenal, (*Z*)-3-hexenal, (*Z*)-3-hexenyl acetate, and (*Z*)-2-hexenol), monoterpenes (β-linalool and γ-terpinene), sesquiterpenes ((*E*)-β-caryophyllene, α-humulene, and β-elemene), and methyl salicylate in tomato plants (Zebelo et al., 2014). These effector compounds not only trigger the defense responses of the host plant but also induce responses that can attract predators of the pest. A study in potato plants, showing that the treatment with volicitin, regurgitant from the insect larvae, and MeJA treatment increased the attraction of Colorado potato beetles, *Leptinotarsa decemlineata* (Say), in comparison to the mechanically damaged potato plants even after 24 h (Landolt et al., 1999). This indicates that the HIPVs produced in response to the effector molecules secreted by the insect pests may also increase the attractiveness of the host plant to the same/other pests.

*Tuta absoluta* (Lepidoptera: Gelechiidae), a major pest of Solanaceae crops, has shown differential behavioral responses to VOCs emitted by the tomato and potato plants in their natural conditions (Caparros Megido et al., 2014). According to the report, enhanced emission of monoterpenes (α-pinene, sabinene, myrcene, δ-2-carene, α-phellandrene, δ-3-carene, and β-phellandrene) was found to be attractive for the pests in tomato plants and enhanced emission of sesquiterpenes (β-caryophyllene, (E)-β-farnesene, germacrene-D, and germacrene-D-4-ol) was found to be attractive in potato plants. However, *T. absoluta* females did not show any preference for oviposition according to these volatile cues. Floral volatiles from tobacco, including (E)-β-ocimene, octanal, (Z)-3-hexenyl acetate, (Z)-3-hexen-1-ol, nonanal, (Z)-3-hexenyl-2-methyl butyrate, decanal, linalool, and (E)-β-caryophyllene, have been found to be attractive for another chewing herbivore, *Helicoverpa assulta* (Guene´e) (Lepidoptera: Noctuidae) (Sun et al., 2012). The attraction of the pest *Phthorimaea operculella* to tobacco plants mediated by GLVs has also been reported by Xiang et al. (2020). Interestingly, geraniol, a monoterpene volatile has been found to deter the oviposition of shoot and fruit borer on eggplant (Ghosh et al., 2022).

Qualitative and quantitative differences in the emitted volatiles have been observed in the tomato plants infested with *T. absoluta* (Silva et al., 2017). Headspace analysis of these plants has shown a consistent association with the emission of fatty acid derivative compounds, including 3-methyl butan-1-ol, (Z)-2-penten-1-ol, (Z)-3-hexen-1-yl-formate, (Z)-2-penten-1-yl butyrate, and few other related compounds. An almost 10-fold increase in the emission of volatiles, including terpenes, aromatic compounds, and fatty acid esters, has been observed post-infestation. A recent metabolome and volatilome analysis of eggplant and tomato post-infestation revealed that differential accumulation of both metabolites and VOCs was responsible for the pest resistance in eggplants (Chen et al., 2021). Interestingly, the study also reported that the borer showed differential behavioral responses during pre-and post-infestation in both eggplants and tomatoes. Terpenes (nerolidol, beta-Cyclocitral, 1,3-cyclohexadiene-1-carboxaldehyde, 2,6,6-trimethyl, and beta-iso-methyl ionone) and a few other ketones, heterocyclic esters, aldehydes, and alcohols emitted from tomato plants could have attracted and/or stimulated the attack of pests, while emission of nerolidol (terpene), 1,3-cyclopentadiene, 5,5-dimethyl-1,2-dipropyl (olefin), and 2-butenoic acid, 3-hexenyl ester (ester) due to mechanical damage or borer infestation from eggplants could have repelled the pest or decreased its survival (Chen et al., 2021). Among the other metabolites, two deterrent compounds, *viz.*, flavonoid compounds (6-hydroxy kaempferol-3-O-rutin-6-O-glucoside) and quercetin-3-O-apiosyl (1 → 2) galactoside), were found to be produced in higher quantities in eggplants. These flavonoids have been shown to modulate the oviposition and feeding of herbivores in different crops (Simmonds, 2001). The induction or enhanced emission of VOCs during the attack of chewing herbivores has also been reported in maize, rice, cotton, and legume crops (Leitner et al., 2005; Sobhy et al., 2017; Qi et al., 2018; Arce et al., 2021).

Recently, GLVs have been shown to emit immediately because of the mechanical damage caused during herbivory in plants, including tomatoes, potatoes, lima beans, *Arabidopsis*, and even trees (Meents and Mithöfer, 2020). Farag and Paré (2002) explained how C_6_ GLV, (*E*)-2-hexenal triggers the defense responses, including the emission of VOCs in tomatoes. Similar reports of induction of defense through mechanical damage in cotton leaves and *Arabidopsis* have also been reported (Rodriguez-Saona et al., 2003, Yamauchi et al., 2018; Arce et al., 2021). The effects of damage-induced volatiles seem to be contradictory in a few plants even among Solanaceae family. Studies by Landolt et al. (1999) reported that mechanical- damage-induced VOCs did not attract Colorado potato beetles significantly in comparison to the VOCs released in response to effector molecules released by larvae feeding. Treatment with *cis*-3-hexenyl acetate (*z*3HAC), another GLV often grouped under DIVs, did trigger defense responses in *Capsicum annuum* (Freundlich et al., 2021). These reports are in line with a well-known fact that damage helps in the recognition of attack by plants but then is not completely sufficient to trigger the full plant defense (Heil, 2009; Fürstenberg-Hägg et al., 2013; Acevedo et al., 2015).

### 3.2 HIPVs during attack of mesophyll-feeding stylet feeders

Mesophyll-feeding stylet feeders include mites and thrips. These insects can empty the cell contents without causing more damage to cell walls and plasma membranes. They primarily feed on the leaf tissues *via* their stylet penetrating the stomatal openings or the intercellular spaces of cells located on tissue surfaces (Bensoussan et al., 2016). Most of the studies have focused on the emission of VOCs from plants under attack by chewing herbivores, while limited reports exist on the emission of HIPVs from plants under attack by other types of feeding herbivores, including mesophyll-feeding stylet feeders (Delphia et al., 2007). Two-spotted spider mite (*Tetranychus urticae*) is a generalist pest that infects almost 150 crop species, including those from the Solanaceae family. Upon infestation with these mites, a susceptible line of chili showed increased emission of VOCs, specifically, (E)-2-4-Hexadiene and methyl salicylate, while the resistant line showed enhanced emission of sesquiterpene compounds. The emission of benzenoid volatiles was found repressed in both the lines (Zhang et al., 2020). In tomatoes, the infestation of *T. urticae* induced the emission of terpenoids and methyl salicylate mediated by JA (Ament et al., 2004). Contradictorily, in a different tomato cultivar under the attack of *T. urticae*, enhanced emission of terpenoids, methyl salicylate, and methyl benzoate induced *via* salicylic acid (SA) signaling was observed (Weinblum et al., 2021). Time course profiling of VOCs emitted from tomato leaves upon spider mite infestation showed the enhanced emission of methyl salicylate, 4,8,12-trimethyltrideca-1,3,7,11-tetraene (TMTT), trans-β-ocimene, trans-nerolidol, and linalool, which relatively kept increasing up to 5 days (Kant et al., 2004). Secondary metabolites, including anthocyanins, were induced in tomato plants almost 5 days after the infestation, thus suggesting that the emission of volatiles is part of the secondary response when mites infect the plants. Delayed emission of terpenoid compounds upon spider mite infection almost after 5 days has also been reported in lima beans and cucumber (Bouwmeester et al., 1999; Mercke et al., 2004). In the case of chewing herbivores, which cause more damage to the foliar tissues, emission of GLVs almost happens immediately in comparison to the VOCs emitted by the plants infected by mites. Another specialist herbivore, i.e., the tomato red spider mite *Tetranychus evansi*, showed similar attractiveness to the foliar volatiles emitted by cultivated African nightshade plants (*Solanum sarrachoides* Sendtner, *S. villosum* Miller, and *S. scabrum* Miller) and VOCs (unsaturated fatty acids) from glandular trichomes of one of the plants studied, which deterred the oviposition of the pest (Murungi et al., 2016). JA-mediated induction of linalool synthase in trichomes of the leaf tissues during spider mite infestation has been reported in tomato (van Schie et al., 2007). Furthermore, Falara et al. (2014) identified a class of geranyl linalool synthases in the Solanaceae family and other angiosperms that are responsible for the production of defensive compounds. They also reported that the corresponding genes can be expressed by treatment with MeJA.

Western Flower Thrips, Onion thrips, and melon thrips have also been shown to prey on the Solanaceae crops. Responses of western flower thrips (*Frankliniella occidentalis*) to plant volatiles when analyzed led to the identification of attractant and repellant VOC molecules for the pest. p-Anisaldehyde, nerol, ethyl nicotinate, and (E)-β-farnesene were found to be attractive at several concentrations, while salicylaldehyde, a benzenoid compound, was found to be repellent for the thrips (Koschier et al., 2000). One of the early reports describing the emission of induced VOCs by white flower thrips feeding was by Delphia et al. (2007) in tobacco plants. VOCs, when analyzed after 4 days of a high level of infection, showed the presence of terpenoid compounds, including (E)-β-ocimene, linalool, β-caryophyllene, unidentified sesquiterpene, farnesene, and nicotine, consistently. Pest attack by western flower thrips in tomatoes has shown a delayed increase in the production of terpenoid compounds (α-pinene, δ-carene, α-phellandrene, α-terpinene, limonene, and β-phellandrene) *via* JA regulatory pathway by increasing the trichome density in leaf tissues of the plant (Escobar-Bravo et al., 2017; Chen et al., 2018).

### 3.3 HIPVs during attack of phloem-feeding stylet feeders

Phloem-feeding stylet feeders include aphids and whiteflies often grouped as sucking pests. They cause minimal damage to the mesophyll cells and rather cause a shift in the plant’s source to sink flow (Kant et al., 2009). One of the earliest report of the production of HIPVs upon aphid (*Myzus persicae* (Sulzer)) attack was in *Solatium berthaultii* Hawkes, which released (*E*)-β-farnesene, a sesquiterpenoid compound from glandular hairs of the shoot system (Gibson and Pickett, 1983). Interestingly, this compound is also believed to be an aphid alarm pheromone (Bowers et al., 1972). Harmel et al. (2007) also reported the changes in the emission levels of terpene, (*E*)-β-farnesene, upon infection of potato plants with *Myzus persicae*. Consistent emission of few fatty acid derivatives (hexanal, E-2-hexenal, Z-2-hexenal, and their alcohols, decanal and phthalic acid, and cis-hexen-1-ol), terpenoids (α-pinene, β-ocimene), and a benzenoid compound (methyl salicylate) during aphid attack has been reported in eggplant, chili, and tomato (Digilio et al., 2012; Cascone et al., 2015; Ali et al., 2022). Consistent emission of limonene and (*E*)-β-farnesene from *Rhopalosiphum padi* (Hemiptera: Aphididae)- infected rice has been reported to increase the resistance of crops against the pest (Sun et al., 2017). Attack of *Lipaphis erysimi* in *Arabidopsis* plants showed enhanced emission of HIPVs when the intensity of attack increased within 24 h. A few volatiles detected include limonene, α-terpineol, benzaldehyde, phenylacetaldehyde, and decan-3-ol (Lin et al., 2016). The emission of methyl salicylate has also been reported from different crops under the attack of aphids (Zhu and Park, 2005).

Whiteflies are another class of sucking pests that attack major crops worldwide. They not only suck up the nutrients from the plant tissue but also promote the growth of pathogenic fungi and act as vectors for several viruses (Perring et al., 2018). *Bemisia tabaci* (Gennadius) and *Trialeurodes vaporariorum* Westwood are two prominent pests affecting several crops worldwide. The production of a sesquiterpene zingiberene (Antonious and Kochhar, 2003) and a fatty acid derivative (Fridman et al., 2005) by glandular trichomes of wild tomatoes have shown deterrence to the infection of the pest. The study by Bleeker et al. (2009) also showed that monoterpene p-cymene was putatively repellent and revealed two additional candidates, i. e., α-terpinene and α-phellandrene, against the pest in tomato lines. Emission of Z-3-hexen-1-ol, α-pinene, α-humulene, (E)-β-caryophyllene, methoxyphenyl oxime, azulene, and 1,1-dimethyl-3-methylene-2-vinylcyclohexane was shown to increase in egg plants infested by greenhouse whitefly, *Trialeurodes vaporariorum* Westwood (Darshanee et al., 2017).

**Figure 2:**
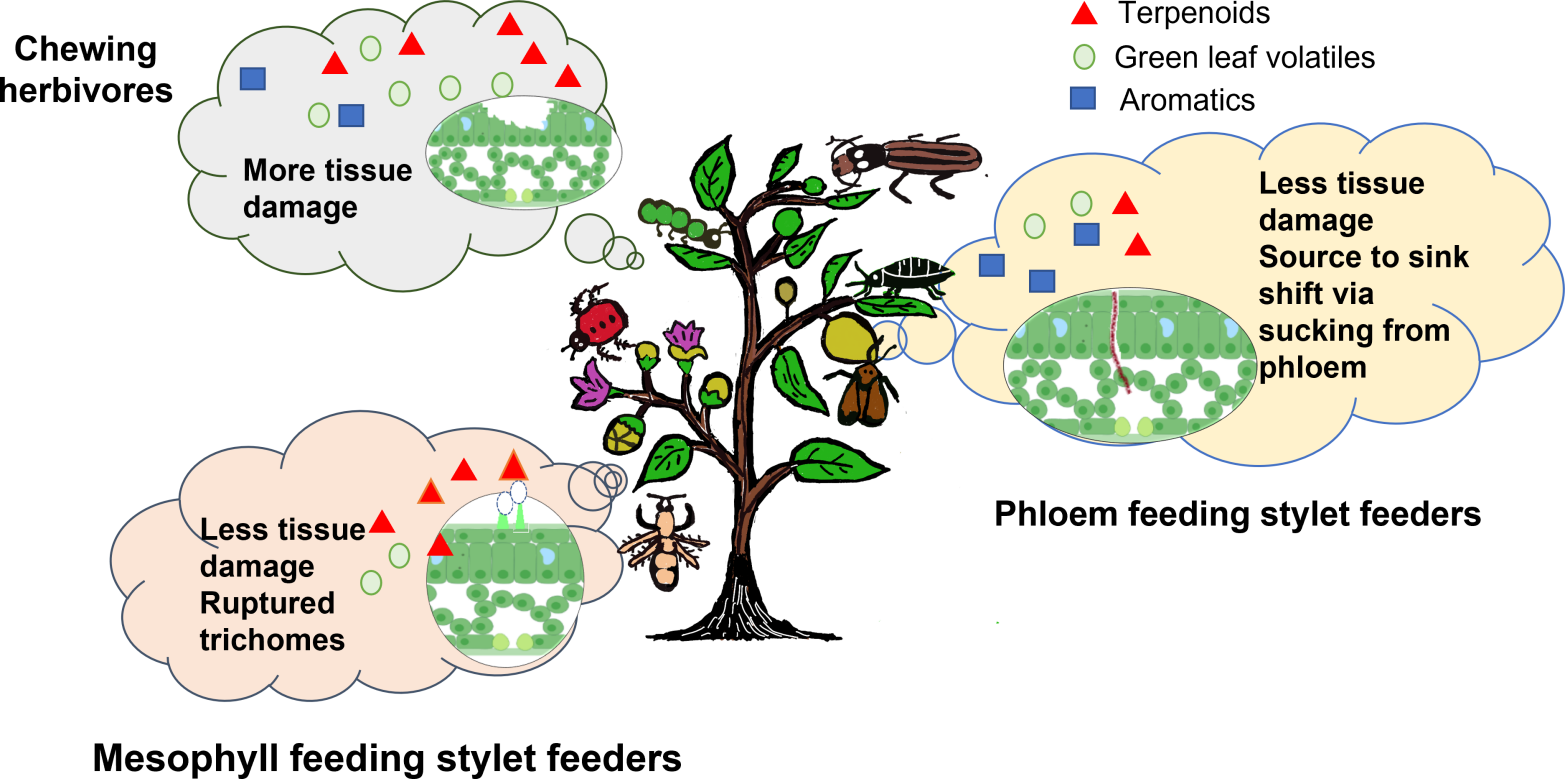
Diversity of HIPVs emitted in Solanaceae crops under herbivory. Emission of higher amounts of VOCs including terpenoids, GLVs, and few aromatics under the attack of chewing herbivores and mesophyll-feeding stylet feeders along with selective emission of aromatics, terpenoids, and few GLVs under the attack of phloem feeding stylet feeders is shown. In set in the figures shows a representative cross-section of leaf tissue damage under respective herbivory.

To summarize, feeding by chewing herbivores induces the emission of GLVs, terpenes (monoterpenes, sesquiterpenes, and homoterpenes), and, at times, methyl salicylate (limited reports) due to the mechanical damage caused to the tissues, released effector molecules, and the eggs laid by the pests on the Solanaceae crops (**Table 1**). Mesophyll-feeding stylet feeders have been shown to induce the emission of terpenoids, and fatty acid esters present in the trichomes of the leaf tissue *via* JA signaling. Very few reports are available on the effector molecules released by mites/thrips inducing defense responses in the host plants (Steenbergen et al., 2018). Phloem-feeding stylet feeders causing minimal damage to the plant tissues release HIPVs like phenylpropanoids/benzenoids (methyl salicylate, benzaldehyde), terpenes (E-β-farnesene), and fatty acid esters (**Figure 2**). Leaf-chewing herbivores are known to activate the JA signaling pathway. While phloem-sucking pests activate the SA signaling pathway at times, even by switching off the JA signaling pathway (Clavijo McCormick et al., 2012). A recent meta-analysis of 236 experiments dealing with HIPVs released by chewing herbivores vs. sucking pests reported higher total amounts of volatiles, including GLVs and terpenoids being released by the chewing herbivores as compared to the sucking pests that selectively induce fewer compounds belonging to benzenoid/phenylpropanoid and terpenoid classes (Rowen and Kaplan, 2016). Differential emission of volatiles when two different types of pests are attacking the same crop plant has also been studied in the Solanaceae family (Gosset et al., 2009; Silva et al., 2017). Davidson-Lowe and Ali (2021) also studied the VOCs emission pattern in potato plants under the co-occurrence of chewing and sucking pests. The study reported that sucking pests preferred uninfected plants over the plants that were already infected with the Colorado potato beetle. Analysis of the emission of HIPVs by plants under different biotic stress conditions can help in identifying the volatile signatures, which may be used for early detection of pest attacks, thereby reducing the crop losses incurred. This review being limited to a few important agricultural crops of the Solanaceae family may have overlooked any other classes of VOCs emitted upon herbivory. In the following sections, we further discuss the dynamic role of plant volatiles affecting direct and indirect defense mechanisms in plants.

## 4 Multifunctionality of HIPVs in plant defense

Plants emit specific blends of info chemicals upon mechanical tissue damage and herbivore attack. HIPVs act as physiological regulators that can prime plant defenses (Hu et al., 2021). Faster and stronger activation of plant defenses in HIPV-exposed plants leading to enhanced resistance in subsequent herbivore attacks is known as “defense priming” (Engelberth et al., 2004; Kim and Felton, 2013). HIPVs prime JA accumulation by modulating the early defense signaling components and subsequently regulating the transcription of jasmonate-responsive genes (Engelberth et al., 2013; Erb et al., 2015). Jasmonates are known to be the key regulators of plant defense and herbivore resistance. Therefore, it is assumed that HIPVs increase plant resistance by regulating the JA signaling pathway. The intricate molecular mechanisms underlying the volatile-mediated defense priming have been explored in several plants and discussed below.

### 4.1 Regulation of GLVs in response to herbivory

**Figure 3:**
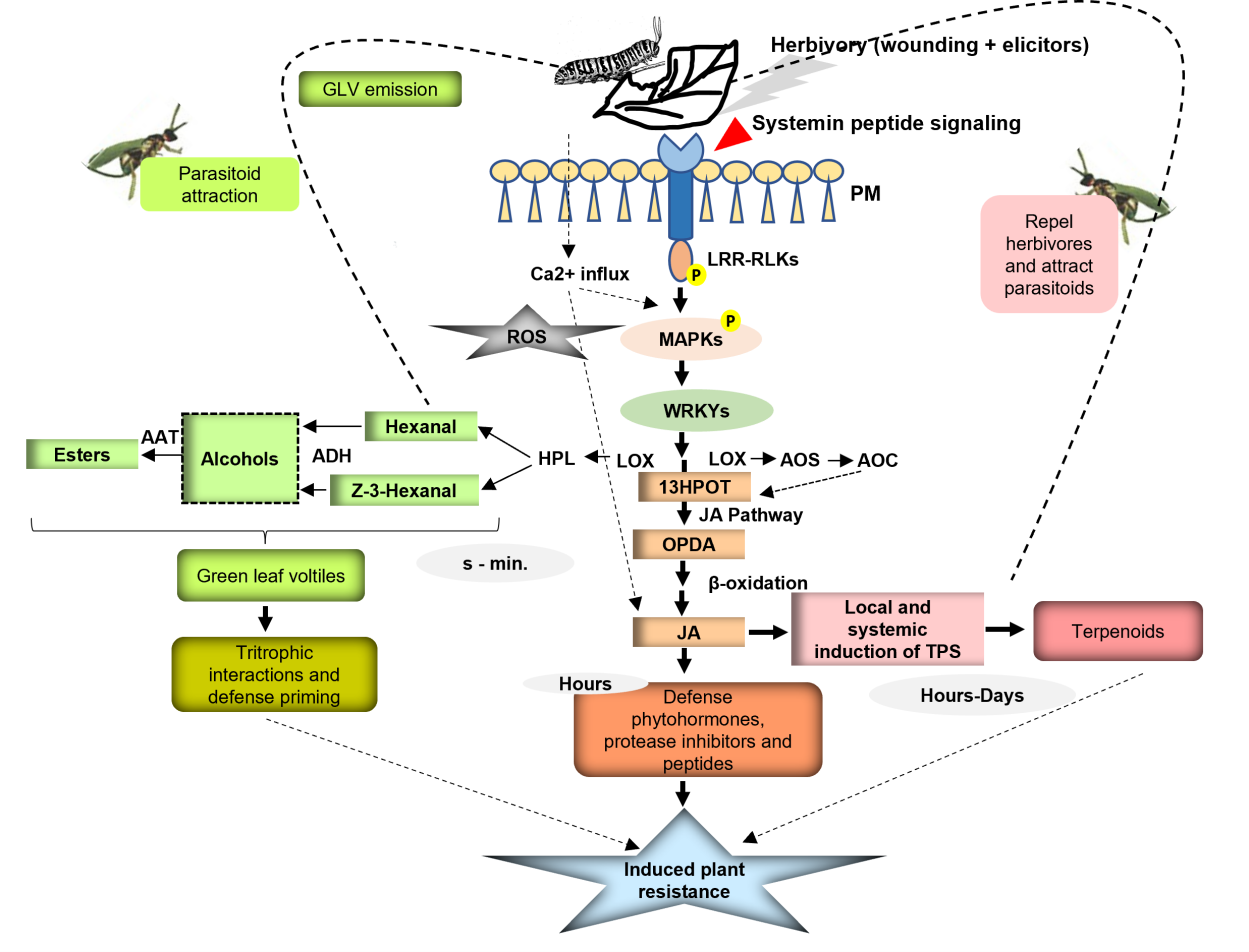
Schematic model of herbivore-induced defense signaling in plants. Role of specific early defense regulators and components comprising of Ca^2+^, LRR-RLKs, MAPKs, WRKY transcription factors, and jasmonate biosynthesis genes leading to downstream defense gene expression to activate plant defenses is illustrated. The role of GLVs and terpenoids in mediating the direct and indirect responses *via* tritrophic interactions is also exemplified.

GLVs, namely, six-carbon aldehydes ((E)-2-hexenal), alcohols ((Z)-3-hexenol), and their esters ((Z)-3-hexenyl acetate) are dramatically emitted by plants upon tissue damage or herbivore attack (Engelberth et al., 2013; Ameye et al., 2017). GLVs being produced from existing precursors are produced very rapidly upon tissue damage. They are produced *via* the lipoxygenase (LOX) pathway, the first product (Z)-3-hexenal being formed by the oxygenation of linolenic acid catalyzed by LOX. The isomerization of (Z)-3-hexenal yields (E)-2-hexenal, which is directly toxic for the infesting herbivores. C_6_ aldehyde forms are converted into corresponding C_6_ alcohols by alcohol dehydrogenases followed by the action of acetyltransferases to form esters (D’Auria et al., 2007). The intermediate product in this pathway, linolenic acid 13-hydroperoxide (13HPOT), also serves as a precursor for JA, which regulates the production of herbivore-induced volatile terpenoids in damaged and undamaged tissues. Hence, GLV and JA bio syntheses both support the effective regulation of HIPV production by plants upon herbivory (Arimura et al., 2009) (**Figure 3**). GLVs are released within seconds of tissue damage from leaves and stem. Real-time volatile analysis studies in *Arabidopsis* have shown peaking of (Z)-3-hexenal at 30–45 s, and alcohol and ester form at 5 min following the damage (D’Auria et al., 2007). It is assumed that (Z)-3-hexenal is the predominant product at the tissue damage site, while alcohol and acetate are formed in the vicinity of the wounded site owing to the ample supply of (Z)-3- hexenal from directly disrupted tissues. Presumably, NAD(P)H and acetyl-CoA from healthy leaves support the production of alcohols and acetates, respectively. Many studies have suggested the spatial differentiation of GLVs between local and distal sites of herbivore damage or mechanical tissue disruption. For instance, in maize and cotton leaves, acetate forms were significantly emitted from the distal sites of herbivory or when treated with MeJA. However, emission of all the GLVs with local and distal differentiation was observed when leaves were artificially damaged or subjected to herbivory (Farag et al., 2005; Arimura et al., 2009). The detailed mechanism leading to the rapid release of hexenal after herbivory or wounding is not yet clarified, but high GLV emissions also upon photooxidative stress have suggested that oxidative damage of membranes could be the primary cause of induction of GLV emissions. Oxidative stress is also considered one of the consequences of herbivore damage (Mithöfer et al., 2004; Mithöfer et al., 2005). GLVs are released immediately at the site of tissue damage and result in moderate plant response specifically, priming the neighboring plants against impending herbivory by inducing the chemical defenses. The induction of several plant- defense-related genes that are induced upon MeJA treatment has been observed upon treatment of *Arabidopsis* plants with C_6_ volatiles— phenylpropanoid- related genes such as phenylalanine ammonia-lyase, chalcone synthase, dihydro flavonol reductase, and LOX pathway genes, including LOX and allene oxide synthase (AOS) (Bate and Rothstein, 1998).

### 4.2 Regulation of volatile terpenoids in response to herbivory

Terpenoids, the structurally diverse natural compounds, are produced in plants *via* two biosynthetic pathways: i) the mevalonate (MVA) pathway in the cytoplasm and ii) 2- C -methyl- D -erythritol 4-phosphate (MEP) pathway in the plastids (Arimura et al., 2009; Nagegowda and Gupta, 2020). Both pathways are independent of each other but produce the same intermediate, the five-carbon compound isopentenyl diphosphate (IDP). Substantial contribution of IDP from the MEP pathway has been reported towards total sesquiterpene biosynthesis upon herbivory (Bartram et al., 2006). The structural diversity of terpenoids is brought about by the terpene synthases (TPSs), which can utilize different prenyl diphosphates as substrates to synthesize hemeterpenes (C_5_), monoterpenes (C_10_), sesquiterpenes (C_15_), homoterpenes (C_11_ and C_16_), and diterpenes (C_20_). Hence, TPSs significantly contribute to the plasticity of terpenoid blends produced in response to herbivory (Arimura et al., 2008a). JA and its precursors act as master switches for upregulating a specific set of defense genes for terpenoid production. As a direct response to mechanical tissue damage mimicking herbivory, JA accumulation is observed locally leading to immediate upregulation of the ocimene synthase gene in lima bean leaves (Arimura et al., 2008b). Gene expression studies have shown the upregulation of terpene synthase genes *TPS7* (encoding for monoterpene, β-ocimene) and *TPS12* (encoding for (E)-β-caryophyllene) in tomato plants damaged by caterpillars (Zebelo et al., 2014). However, the release of *de novo* synthesized terpenes from plants may take several hours to days (Turlings et al., 1998; Danner et al., 2015).

The synergistic and antagonistic crosstalk among the influx of calcium ions, JA, and ethylene signaling regulates terpenoid biosynthesis. Plants show varied responses to sucking arthropods and chewing insect pests. In plants damaged by sucking arthropods, the specific terpenoid blend is regulated by the antagonistic crosstalk of SA with JA, while chewing herbivores stimulate the calcium influx, which acts as a secondary messenger for the activation of the JA signaling pathway (Arimura et al., 2008a). Generally, negative interactions between JA and SA signaling have been reported, but this crosstalk can show variations depending upon the degree of damage, timing, and the specific herbivore leading to huge differences in the blend of terpenoids being emitted. Ethylene plays a role in modulating the early signaling events like cytoplasmic Ca^2+^ influx and downstream JA-dependent biosynthesis, which in turn can regulate terpenoid production (**Figure 3**). Moreover, chemical elicitors like fatty acid-amino acid conjugates (FACs) present in oral secretions of lepidopteran larvae synergistically induce plants to release HIPVs (Halitschke et al., 2001; Yoshinaga et al., 2010). Oral secretions (OS) from fall armyworm, *Spodoptera frugiperda*, have shown the presence of proteolytic fragments Inceptin [+ ICDINGVCVDA −] and the related peptides [+ (GE) ICDINGVCVDA −] eliciting rapid production of JA, SA, and ethylene (Schmelz et al., 2007). β-Glucosidase, volicitin, and caeliferins are other reported insect oral elicitors known for inducing the production of HIPVs. Contrary to this, very little is known about oral elicitors from sucking arthropods except for oligogalacturonides, which are assumed to induce Ca^2+^ influx due to cell wall digestion (Will and van Bel, 2008).

### 4.3 Regulation of early defense signaling and downstream gene expression

The molecular mechanism of integration of HIPVs into early defense signaling leading to JA accumulation and subsequent defense gene expression remains elusive demanding more exploration. However, the role of specific early defense regulators and components has been contemplated based on several research studies. The first event after the damage of tissues by chewing caterpillars is the influx of cytosolic Ca^2+^ owing to plasma trans-membrane potential (Vm) depolarization (Asai et al., 2009; Zebelo et al., 2012). Exposure to volatiles such as GLVs has shown increased cytosolic Ca^2+^ flux in tomato and *Arabidopsis* plants. The rise in cytosolic calcium ions is also accompanied by a burst of reactive oxygen species (ROS), including hydrogen peroxide (H_2_O_2_) and nitric oxide (NO) (Maffei et al., 2006). These events occurring within seconds to hour(s) of insect damage are considered as early plant defense responses. Ca^2+^ binding proteins like calmodulins and Ca ^2+^ -dependent protein kinases further integrate the signal to mitogen-activated kinases. Ye et al., 2019 reported direct induction of leucine-rich repeat receptor-like kinase genes (*OsLRR-RLK1*) in rice upon exposure to indole, a commonly emitted HIPV. This study also reported subsequent priming of mitogen-activated protein kinase (*OsMPK3*) and the WRKY transcription factor gene (*OsWRKY70*) for stronger expression leading to jasmonate accumulation and herbivore resistance (Ye et al., 2019). It was concluded that LRR kinases have an upstream function in perceiving the HIPVs. The activation of receptor-like kinases and apoplastic O_2_^−^ and H_2_O_2_ production are supposed to be linked, but the exact connection between these links is still not clear. H_2_O_2_ accumulation is known to upregulate the genes that encode for antioxidative enzymes like superoxide dismutase, catalase, ascorbate peroxidase, glutathione reductase, and monodehydro ascorbate reductase under biotic and abiotic stress (González-Bosch, 2018). It is also anticipated that it may have specific transcription factor targets within the WRKY gene family. In maize, pre-exposure to GLVs has shown direct increased expression of *ZmMAPK6* and *ZmWRKY12* genes (Engelberth et al., 2013). These findings suggest that GLVs can directly induce defense genes and strengthen the jasmonate signaling pathway. Certainly, *OsMPK3*, *OsWRKY70*, and JA form a signaling cascade and positively regulate the resistance to chewing herbivores in plants (**Figure 3**). In addition, a clear role of reactive oxygen species in the early perception and signaling to activate plant defenses is established. Experiments with mutant plants deficient in JA biosynthesis/signaling have also validated that a functional jasmonate signaling pathway is required for HIPV-mediated defense priming in plants (Ye et al., 2019). Therefore, changes in the transcript level of defense-related genes and production of subsequent metabolites, including terpenoids, occur hour(s) to day(s) after the insect damage and comprise the late plant defense responses.

Alternate mechanisms for the molecular basis of VOC recognition have been suggested by a few studies advocating a prominent involvement of transcriptional co-repressors bound to VOCs in regulating the gene expression in plant cells (Nagashima et al., 2019). Direct binding of caryophyllene analogs (sesquiterpenes) to TPL-like proteins (encoded by *NtTPLs*) and dose-dependent responses upon overexpression were observed *in vitro* and *in vivo* in tobacco BY-2 cells and tobacco plants, respectively (Nagashima et al., 2019). Hence, a dual role of TPLs as co-repressors for JA-mediated signaling and as VOC-binding proteins have been proposed, which acts upstream of other transcription factors. However, to generalize this finding, more studies with other VOCs need to be performed. Nevertheless, such reports lead to suggest that plants use VOC-sensing mechanisms *via* nuclear proteins and not membrane-bound receptors. Therefore, the exact mechanism of sense that leads to transcriptional regulation of defense genes is an appealing topic to be explored.

Defense-related enzymes like peroxidase (POD), polyphenol oxidase (PPO), and lipoxygenase (LOX) show significantly higher expression in plants damaged by caterpillars or aphids or mechanically injured plants treated with insect oral secretions (Zebelo et al., 2014; Pingault et al., 2021). All three enzymes are components of the octadecanoid signal pathway, which regulates JA production (Felton et al., 1989). LOXs catalyze the oxidation of linolenic acid in the JA signaling pathway. PODs and LOXs are anti-oxidative enzymes that limit the nutritional quality of the plants to the insect herbivores, thereby increasing the resistance (Felton et al., 1992). PPO is also an inducible enzyme known for its defensive role against insect herbivores and pathogens (Zebelo et al., 2014). In succession to high POD activity, upregulation of several genes, including proteinase inhibitors, has been reported in tomatoes and *capsicum* upon herbivory by caterpillars or treatment with insect oral secretions (Damle et al., 2005; Mishra et al., 2012). In Solanaceous plants, accumulation of proteinase inhibitors (PIs) in the damaged and the undamaged leaves has been reported as one of the major consequences of mechanical wounding or insect herbivory. Generally, the responses to insect feeding have been observed to be more complex as compared to mechanical wounding owing to the elicitors present in insect OS or regurgitant (Hermsmeier et al., 2001; Mishra et al., 2012; Zebelo et al., 2014). Systemin, an 18-amino acid peptide derived from a 200-amino acid precursor prosystemin is known as proteinase inhibitor-inducing factor (Pearce et al., 1991; Orozco-Cardenas et al., 1993) in Solanaceous plants. Systemin has been identified as the systemic signal owing to its phloem mobility, and its treatment shows induced PI accumulation in plants (Narvaez-Vasquez et al., 1994). In addition, coordinated synthesis of immunoregulatory signals like ethylene, hydrogen peroxide, cytosolic calcium ion influx, and plasma membrane depolarization leading to transcriptional reprogramming by systemin has been reported (Ryan, 2000; Kandoth et al., 2007). However, peptide mediator-like systemin has not been identified in other families of plants in the context of ant–herbivore defense. A series of elicitor peptides (ZmPeps) have been reported in maize, which trigger the biosynthesis of herbivory-associated VOCs and also regulate phytohormone biosynthesis and the accumulation of transcripts related to anti-herbivore defense, including proteinase inhibitors (Huffaker et al., 2013; Huffaker, 2015).

### 4.4 Indirect effects of HIPVs during herbivory

HIPVs attract arthropod predators and parasitoids of herbivores acting as an indirect means to repel insect pests (Dicke et al., 1998; Reddy, 2002; Ayelo et al., 2021) (**Figure 3**). Parasitoids use these volatiles as cues to search for their preys. Most of the vegetative volatiles that have been identified in repelling herbivores and attracting herbivore enemies are either terpenes ((E)-β-Ocimene, (E)-β-Caryophyllene, (E)-β-Farnesene), or GLVs (Isoprene) (Unsicker et al., 2009). For example, monoterpene volatiles have been reported to repel the ovipositing females of diamondback moth, and isoprene was shown to deter the tobacco hornworm caterpillars from feeding on the isoprene-releasing transgenic tobacco lines (Laothawornkitkul et al., 2008; Wang et al., 2008). These interactions are specific to each insect–plant interaction. For instance, *Cotesia marginiventris*, a parasitoid of *Spodoptera litura*, gets attracted to *Arabidopsis thaliana* plants emitting (E)-β-farnesene, (E)-α-bergamotene, and other sesquiterpenes (Schnee et al., 2006). The indirect effect is extended to even vertebrate predators of herbivores in a few instances. More attacks from birds were observed on caterpillars attached to infested trees emitting terpenes, specifically, (E)-β-ocimene, linalool, and 4,8-dimethylnona-1,3,7-triene (DMNT) (Mäntylä et al., 2008). While most of the reports on the volatile attraction of herbivore enemies are on the aerial parts of the plants, emissions from roots attracting the nematodes that prey on attacking insect larvae are also known (Rasmann et al., 2005). For example, a root-feeding pest, *Diuraphis noxia*, induces the emission of a monoterpene volatile, 1,8-cineole, which is toxic and acts as a repellant to Coleopteran insect pests (Tripathi et al., 2001). Roots of *Thuja occidentalis* upon attack by Black vine weevils release volatiles that attract the entomopathogenic nematodes (Van Tol et al., 2001). However, the tri-trophic interactions resulting from volatile emissions are a result of the combined effects of above- and below-ground herbivory. Methyl salicylate, a constituent of insect-induced plant volatiles, has been reported to be very effective for the indirect defense of plants by attracting many insect predators and mites and inhibiting the oviposition of the moths (Zhu and Park, 2005; Ulland et al., 2008).

HIPVs mediate the attraction of predators and induce defense responses in neighboring plants (Baldwin and Schultz, 1983; Rhoades, 1983; Turlings and Erb et al., 2018). Maize plants exposed to GLVs from neighboring plants emit increased quantities of sesquiterpenes, thereby activating the direct defenses and attracting an important parasitoid of *S. littura* larvae, i.e., *C. marginiventris* (Ton et al., 2007). Similarly, approximately 30 volatiles including methyl salicylate and methyl benzoate have been reported from rice plants infested with *S. frugiperda*, which collectively result in the attraction of natural enemies of *S. frugiperda* such as *C. marginiventris* (Yuan et al., 2008). The study by Arimura et al. (2001) reported that emitted VOCs from lima- bean-infested plants by *T. urticae* activated the transcription of pathogenesis-related and phenylalanine ammonia-lyase genes in undamaged neighboring plants. Studies also showed where the defense of receiver plants has increased upon exposure to HIPVs in Solanaceae crops (Cascone et al., 2015; Angeles Lopez et al., 2012; Dahlin et al., 2015). A recent study by Abdala-Roberts (2022) has reported a lack of upregulation of insect resistance in receiving potato plants due to exposure to HIPVs from Colorado potato beetle-infested plants (emitter plants). Engelberth et al. (2004) has shown priming of defense responses in maize plants upon exposure to airborne VOCs. Holopainen et al. (2013) discussed the fate of HIPVs once emitted from the host plant. VOCs such as terpenoids, fatty acid esters, and methyl salicylate show different atmospheric lifetimes under different environmental conditions in the presence or absence of contaminants. Furthermore, these VOCs, when up taken or absorbed by diffusion into plant tissues, undergo reduction/oxidation reactions (metabolism), glycosylation, and glutathionylation (Matsui, 2016). Thus, responses induced by them can also vary from plant to plant and environment to environment.

## 5 Metabolomics as a tool to understand HIPVs and plant defense

The importance of plant VOCs are very crucial as signaling molecules in plant defense and plant–plant/plant–insect communication. Plant VOCs are the info chemicals that mediate intra- and interspecific interactions (Dudareva et al., 2013). However, several challenges concerning abiotic conditions, the lifetime of VOCs in different environments, and limitations of performing experiments at the field level to understand the actual effect of HIPVs in inducing defense in crop systems must be addressed. In recent times, metabolomics and transcriptomics analysis have been carried out to understand metabolite networking during the attack of pests especially for identifying metabolites responsible for inducing pest resistance in plants. For example, Macel et al. (2019) have identified diterpene glycosides as defensive compounds in pepper plants against thrips. Similarly, Liu et al. (2022) identified quinic acid as a metabolite that offers resistance against western flower thrips in eggplants. Progress in -omics technologies in recent times have intensified our knowledge of VOCs diversity, genes encoding enzymes that are responsible for their biosynthesis, the regulatory mechanisms involved in their formation, and downstream defense gene expression. Metabolomic analysis, including volatilome studies, must be designed and taken up in the field level studies to gain complete insight into the dynamic plasticity of HIPVs produced, defense responses induced in plants, behavioral changes in pests, allelopathic interactions within the kingdom, and cross-kingdom species. However, several technical challenges do exist in this domain including developing a single sampling method to profile both volatile and non-volatile metabolites (Maag et al., 2015).

## 6 Conclusion and future prospective

In this review, we try to summarize the diversity of HIPVs induced by the attack of different types of herbivores in Solanaceae crops. Furthermore, we highlight the multifunctional role of HIPVs in regulating direct and indirect defense responses in Solanaceae crops and provide a brief overview of upcoming “ omics” driven methodologies in understanding plant–herbivore interactions. Truly, these compounds act as infochemicals during herbivory in plants and mediate several interactions at different trophic levels. Metabolic engineering of floral and defense-related VOCs is a promising approach to enhance plant chemo-diversity and mediate plant–insect interactions to enhance insect resistance in crop plants. The introduction of new gene(s) or upregulation or downregulation of existing biochemical components have been some of the strategies implemented in recent studies. Constitutive overexpression of (E)-β-caryophyllene synthases in rice has shown improved above-ground plant defense by attracting parasitoids (Degenhardt et al., 2009; Xiao et al., 2012), while in maize, significant improvement in below-ground plant defense from root pests was observed on restored emission of (E)-β-caryophyllene (Degenhardt et al., 2009). The production of volatile patchoulol with sesquiterpenes in transgenic tobacco shows deterrence to tobacco hornworms, which otherwise show 20%–50% more damage to the wild-type plants (Wu et al., 2006). Overexpression of strawberry linalool/nerolidol synthase gene (*FaNES1*) in *Arabidopsis* results in high emission of linalool, thereby repelling the aphids, *Myzus persicae* (Aharoni et al., 2003). Transgenic tobacco plants overexpressing yeast acyl-CoA D9 desaturase or the insect acyl-CoA D11 desaturase showed elevated levels of GLV (Z)-3-hexenal, thereby leading to an increased 13-lipoxygenase activity, which regulates the defense pathway (Hong et al., 2004). Heterologous expression of a sesquiterpene synthase gene in *Solanum lycopersicum* (cultivated tomato) showed the production of a novel insecticidal compound that increased resistance to whiteflies and spider mites (Bleeker et al., 2012). Such reports suggest that it is possible to improve the natural plant defense mechanisms by metabolic engineering of VOCs, providing an alternate pest management strategy (Khan et al., 2000; Dudareva and Pichersky, 2008; Zhou and Jander, 2021). However, the impact of altered VOC emissions on insect behavior, effects on other tri-trophic interactions, and overall ecological implications need to be explored to a larger extent to implement these approaches in an agriculture setting. Other significant methodological challenges that VOC engineering encounters are negative effects on plant growth and development owing to the limited carbon availability, the toxicity of the volatile compounds to the non-target organisms, the formation of unpredicted compounds, and no yield of desired volatile due to lack of biosynthetic precursors (Dudareva et al., 2013). A more holistic view of plant metabolic networks is the need of the hour to engineer plant- defense-specialized metabolites to enhance insect resistance. The evolutionary context of HIPVs must also be considered when designing such modified crops. As Dicke and Baldwin (2010) suggested, these HIPVs also function beyond distress signals and rather act as infochemicals in an infochemical web in an ecosystem. Therefore, metabolic engineering to improve plant fitness is a very fruitful area for future research work.

## Author contributions

The authors MM and NK have contributed equally towards collecting, interpreting, analyzing, and writing the manuscript. All authors contributed to the article and approved the submitted version.
